# Successful surgical treatment for aortoenteric fistula after esophagectomy: a case report

**DOI:** 10.1186/s44215-024-00132-y

**Published:** 2024-07-15

**Authors:** Erica Nishimura, Hirofumi Kawakubo, Asano Ryota, Takeuchi Masahi, Satoru Matsuda, Kazumasa Fukuda, Rieko Nakamura, Yuko Kitagawa

**Affiliations:** 1 Department of Surgery, Keio University School of Medicine, Tokyo, Japan; 2 Ogikubo Hosipital, Tokyo, Japan

**Keywords:** Aortoenteric fistula, Esophagectomy, Stent graft repair, Esophageal cancer

## Abstract

**Background:**

An aortoenteric fistula (AEF) is a rare and lethal complication of esophagectomy. Fistulas frequently result from problems regarding acute infections or leaks, which are typically evident weeks after the treatment. However, some cases exhibit AEF years after the initial operation. Here, we describe a rare case of AEF caused by chronic friction of the stapler toward the aortic arch, in which stent graft repair and surgery were successful.

**Case presentation:**

A 71-year-old man had undergone esophagectomy for esophageal carcinoma and reconstruction with a gastric conduit through the posterior mediastinal route 11 years previously. He visited our outpatient clinic with the chief complaint of hematemesis. However, after arrival, he experienced massive hematemesis and severe shock due to bleeding from an AEF. Endoscopic hemostasis using a Sengstaken-Blakemore tube followed by stent graft repair controlled the bleeding. We performed a partial resection of the gastric conduit, including the fistula, followed by digestive reconstruction using a jejunal interposition graft. The patient recovered gradually after receiving intensive care and was discharged 115 days after hospitalization.

**Conclusions:**

We present a rare case of bleeding due to AEF long after esophagectomy, which was successfully treated with endovascular stent graft repair and surgery. Endoscopic hemostasis using a Sengstaken-Blakemore tube followed by stent graft repair was effective.

## Background

An aortoenteric fistula (AEF) is a rare and fatal complication of esophagectomy. Because the reconstructed gastric conduit is surrounded by vital organs such as the heart, aorta, and trachea, penetration of these organs can be serious and fatal [1]. In most cases, fistulas are caused by complications of acute infection or leak, which are usually seen within weeks after the procedure [2]. However, some cases show AEF long after the first surgery.

Here, we report a rare case of AEF suspected to be caused by continuous contact pressure between the linear staple and aorta 11 years after esophagectomy. We successfully controlled bleeding by inserting a Sengstaken-Blakemore (SB) tube, followed by covering the aorta with a thoracic stent graft. The gastric conduit, including the anastomosis and fistula, was partially removed, and digestive reconstruction was performed using a jejunal interposition graft.

## Case presentation

**Fig. 1:**
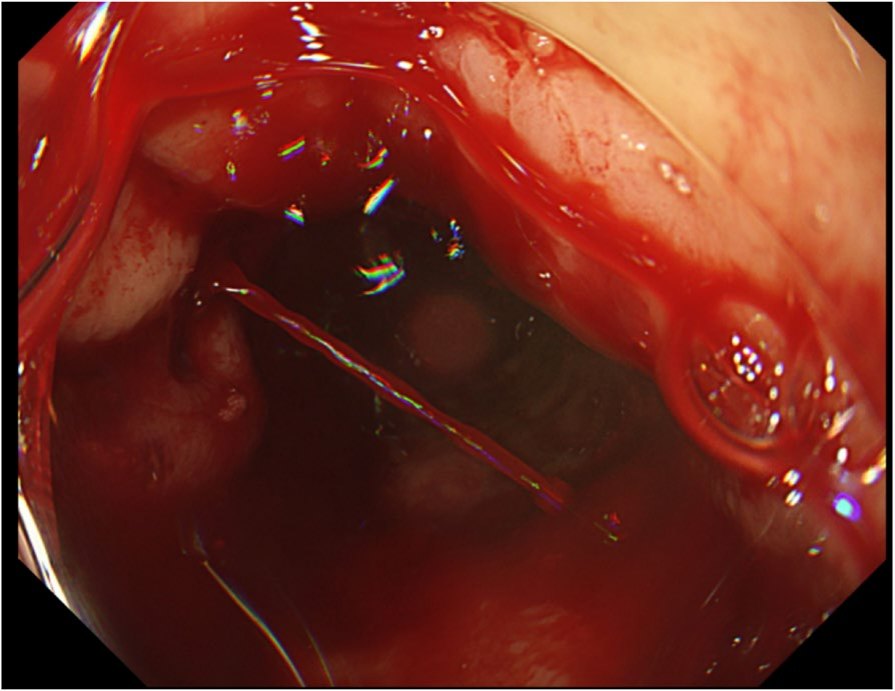
Gastrointestinal endoscopic findings Arterial bleeding from the anterior wall of the gastric conduit has been revealed. **b** Arterial bleeding from the anterior wall of the gastric conduit

**Fig. 2:**
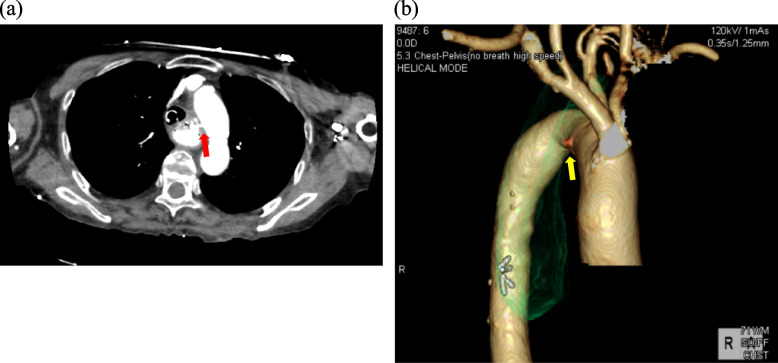
Computed tomography (CT) angiogram showing a fistula between the gastric conduit and the thoracic aorta. **a** Large amount of blood clots occupying the gastric conduit (red arrow). **b** Arterial bleeding from the anterior wall of the gastric conduit (yellow arrow)

A 71-year-old man underwent esophagectomy for esophageal carcinoma 11 years ago. Surgery was performed using video-assisted thoracoscopic esophagectomy with three-field lymphadenectomy for esophageal carcinoma together with the cervical esophageal anastomosis with a gastric conduit through the posterior mediastinal route. He was diagnosed with a recurrence of the right recurrent nerve lymph nodes 6 months after surgery and received chemoradiotherapy. Since then, he showed no recurrence over the next 10 years following the operation. He visited our outpatient clinic with a chief complaint of a small amount of hematemesis. However, he suddenly experienced massive hematemesis and severe shock, with a blood pressure of 56/33 mmHg and a pulse rate of 144 bpm. Blood tests revealed a hemoglobin level of 4.4 g/dL (at the lowest level). The other laboratory results are shown in Table 1. With the use of blood transfusion and vasopressor support, we conducted gastrointestinal endoscopy. Gastrointestinal endoscopic examination revealed a large amount of blood clots and arterial bleeding from the anterior wall of the proximal part of the gastric conduit (Fig. 1). No peptic ulcers were observed at the bleeding site. Because the bleeding was exclusive, we suspected an AEF and inserted an SB tube to stop the hemorrhage. His vital signs gradually recovered, and computed tomography (CT) was performed. To identify the bleeding spot, we carefully deflated the balloon under strict surveillance during the arterial phase, which revealed a fistula between the gastric conduit and thoracic aorta beneath the aortic arch (Fig. 2a, b). The fistula was located on the stapler at the lesser curvature of the gastric conduit. Emergency endovascular stent graft repair was performed. A covered thoracic stent graft (Medtronic Valiant VAMF3030C100TJ; diameter, 30 mm; length, 117 mm) was inserted. The SB tube was deflated the next day, and hemostasis was confirmed. Broad-spectrum antimicroial agents were used immediately to avoid exposing the stent to the gastric conduit. However, the patient was afebrile, wherein the repeated blood cultures showed negative results throughout the clinical course. We planned to resect the gastric conduit immediately after his recovery to reduce the risk of stent infection.

**Table 1:** Laboratory tests on admission

Complete blood count		Blood chemistry	
WBC	3300 /μl	AST	13 U/L
RBC	1.42×10^6^ /μl	ALT	6 U/L
Hb	4.4 g/dL	LDH	196 U/L
Ht	13.3 %	ALP	46 U/L
Plt	41 ×10^3^ /μl	γ-GTP	20 U/L
		UN	47.1 mg/dL
		Cre	0.94 mg/dL
		Na	137.3 mEq/L
		K	4.0 mEq/L
		Cl	104 mEq/L
		CRP	0.06 mg/dL

**Fig. 3:**
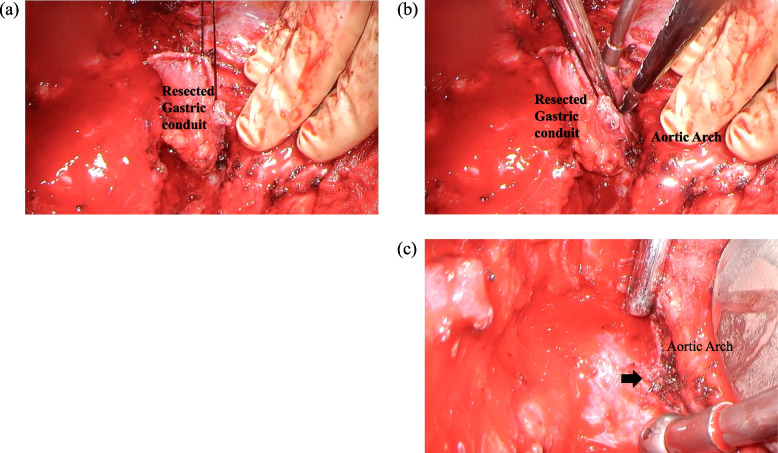
Surgical findings following the resection of the thoracic part of the gastric conduit. **a**, **b** Extracting the stump of the gastric conduit from the aortic arch. The adhesion of the gastric conduit and the aortic arch was strong. **c** After resecting the gastric conduit. The fistula was covered with the remnant stapler (arrow)

Within 19 days after endovascular stent graft repair, we partially resected the gastric conduit in the posterior mediastinal route, including the anastomosis and fistula, and placed the remnant gastric conduit at the intrathoracic subcutaneous portion. Finally, we created a cervical esophageal stoma and jejunal fistula for enteral nutrition. Because the aortic arch and the gastric conduit were strongly attached and the stapler at the lesser curvature of the gastric conduit was exposed at the same site, we suspected that the chronic friction of the stapler toward the aortic arch caused the fistula (Fig. 3a–c). The aortic stent was not exposed during surgery, and the fistula was covered with a remnant stapler.

**Fig. 4:**
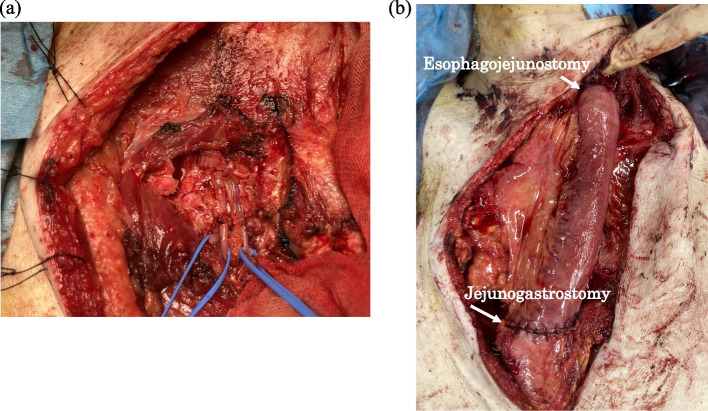
Digestive reconstruction using jejunum interposition. **a** The isolated right internal mammary artery and vein. **b** Intraoperative photograph after reconstruction

The patient recovered gradually after receiving intensive care. Forty-two days after surgery, digestive reconstruction was performed using a jejunal interposition graft through the antethoracic route. First, we mobilized the esophageal stump and the remnant gastric conduit to prepare for the reconstruction of the digestive tract. We used the second jejunal vessels as donor vessels for the free jejunal flap. Plastic surgeons performed revascularization using the right internal mammary artery and vein. Later, esophagojejunostomy and jejunogastrostomy were performed (Fig. 4a, b). Antibiotics were administered until the reconstruction. The patient was discharged 115 days after hospitalization. Blood culture results were negative throughout the clinical course.

## Discussion and conclusions

In many cases, bleeding from AEF has been reported to be fatal when the diagnosis was made [3]. However, in recent years, the development of endoscopic hemostatic devices and rapid endoscopic treatment strategies have enabled us to achieve effective primary hemostasis. Previously, five cases revealed AEF after esophagectomy, which was successfully treated (Table 2). As for the etiology, AEF was mostly caused by ulceration of the reconstructed gastric conduit. Ulceration is thought to be caused by mechanical damage due to pulsation of the descending aorta or by a weakened mucosal barrier caused by postoperative radiation, infection with *Helicobacter pylori*, and the use of nonsteroidal anti-inflammatory drugs (NSAIDs) [4]. However, Okamura et al. reported a case of ulcer formation while continuously using a proton pump inhibitor, with no evidence of *Helicobacter pylori* infection or use of daily NSAIDs [5]. Therefore, they speculated that the ulceration was caused by the anatomical and physiological characteristics of the reconstructed gastric conduit after esophagectomy. Routine gastrointestinal endoscopy is performed to monitor esophageal cancer recurrence. Therapeutic intervention should be initiated in patients with evidence of gastric conduit ulcers as soon as possible, and they would need great caution when they present with any symptoms of hematemesis. In the present case, there was no evidence of a gastric conduit ulcer. CT showed that the stapler at the lesser curvature of the gastric conduit was close to the aortic arch and descending aorta, and we speculated that chronic friction caused the fistula. This mechanism has been previously described to be responsible for the formation of a tracheogastric fistula [6].

**Table 2:** Previous case reports of aorto-gastric fistula occurring after esophagectomy for esophageal cancer

Author	Age	Sex	Period after esophagectomy	Chief complaint	Etiology	Treatment for the bleeding	Treatment involving the aorta	Clinical outcome
Takebayashi [7]	46	F	7 years	High fever	Ulcer	SB tube	Pericardial patch	Died (18 days)
Okamura [5]	67	F	11 years	Gastric discomfort	Ulcer	Resecting gastric conduit	Directly closed	Survived (6 months)
Wei [8]	59	M	14 months	Gastric discomfort	Ulcer	Stent graft repair	Stent graft repair	Survived (4 months)
Chotai [9]	57	F	15 years	Hematemesis	Ulcer	Stent graft repair	Stent graft repair	Survived (4.5 months)
Sumiya [10]	54	M	46 days	Hematemesis	Minor leakage	Resecting gastric conduit	Directly closed	Survived (22 years)
Present case	71	M	11 years	Hematemesis	Chronic friction	Sengstaken-Blakemore tube	Stent graft repair	Survived (115 days)

The treatment strategy after controlling the bleeding is also debatable, especially for the aortic arch portion. In previous reports, direct suturing or stenting was an option for aortic replacement (Table 2). Inserting a thoracic stent graft could be a promising treatment strategy, as there is no need for open surgery, and definite hemostasis can be achieved. Direct suturing has been reported to cause pseudoaneurysms postoperatively [5], and ruptures may result in sudden death. Therefore, a follow-up CT should be performed on these patients to monitor the development of pseudoaneurysms. On the other hand, there is always a risk of stent infection after graft stent repair. Chotai et al. reported a case in aortic stents had to be removed because of infection [9]. In their case, the ulcerated gastric conduit was not removed after stent replacement; consequently, the aortic stent was visible through the defect in the gastric conduit wall. However, there is also a case that graft stent infection was avoided in the long term. Sumiya et al. reported a patient who survived without infection of the aortic stent for more than 2 decades [10]. In their case, resection of the gastric conduit and direct suturing of the aorta was first performed, and graft stenting was performed because of the presence of a pseudoaneurysm. We decided to resect the gastric conduit and perform a secondary reconstruction surgery without removing the graft stent. This is because repeated thoracotomies were considered highly invasive, as the adhesions of the gastric conduit and aorta were thought to be severe. Particularly in this case: the patient has received chemoradiotherapy due to recurrence of the right recurrent nerve lymph nodes. According to previous reports, chemoradiotherapy itself was found to be a risk factor for AEF [11]. Furthermore, thoracotomies after chemoradiation were associated with an increased risk of pulmonary complications, which may be fatal [12]. We did not perform an aortic arch repair, because there was no evidence of stent infection, and determined that the patient could not tolerate single-lung ventilation for aortic arch repair due to his poor performance status after repeated surgeries.

On the contrary, when AEF was formed near the aortic arch, aortic arch replacement could be considered. Although graft stent could be promising to control bleeding as a primary intervention [13], branched endovascular aortic arch repair in the acute setting was practically difficult due to the lack of a devise [14]. In these cases, total arch repair assisted with the frozen-elephant-trunk technique (FET) [15, 16] may be considered. However, the surgical risk could be extremely high regarding the repeated thoracotomy involving adhesions with the surrounding organs.

In conclusion, we present a rare case of AEF caused by chronic friction of a stapler that tore the aortic arch 11 years after esophagectomy. Endoscopic hemostasis using an SB tube followed by stent graft repair was effective. Although it is difficult to save patients with AEF, a premeditated surgical strategy after successful hemostasis will enable the patient to recover fully.

## Data Availability

The authors confirm that the data supporting the findings of this study are available within the article.

## Abbreviations

AEF: Aortoenteric fistula
SB: Sengstaken-Blakemore
CT: Computed tomography
NSAIDs: Nonsteroidal anti-inflammatory drugs
